# Surface-Specific Caries Preventive Effect of an Intervention Comprising Fissure Sealant, Povidone-Iodine and Fluoride Varnish in a Remote Indigenous Community in Australia

**DOI:** 10.3390/ijerph17062114

**Published:** 2020-03-23

**Authors:** Santosh K. Tadakamadla, Ratilal Lalloo, Jeroen Kroon, Newell W. Johnson

**Affiliations:** 1 School of Dentistry and Oral Health, Griffith University, Gold Coast, Queensland, QLD 4215, Australia; j.kroon@griffith.edu.au (J.K.); n.johnson@griffith.edu.au (N.W.J.); 2 School of Dentistry, The University of Queensland, Herston, Queensland, QLD 4072, Australia; r.lalloo@uq.edu.au; 3 Menzies Health Institute Queensland, Griffith University, Gold Coast, Queensland, QLD 4215, Australia; 4 Dental Institute, King’s College London, WC2R 2LS, UK

**Keywords:** Aboriginal and Torres Strait Islander people, dental caries, fluoride varnishes, pit and fissure sealants, topical fluoride, tooth surfaces

## Abstract

This study evaluates the effect of a topical intervention comprising of fissure sealant, povidone-iodine, and fluoride varnish in preventing caries on occlusal, approximal, and smooth surfaces. This three-year clinical trial was conducted in a remote Indigenous community of Australia. All schoolchildren (age range: 4–17) were invited to participate; those with parental consents to receive three-annual epidemiological examinations and interventions constituted the experimental group, while those with consents for only the epidemiological examination formed a comparison group. The intervention group received an annual application of fissure sealant, povidone–iodine and fluoride varnish for two consecutive years along with the restoration of any cavitated lesions, while the comparison group did not receive any intervention except for the usual care that included emergency treatment and restorations. Incipient and advanced caries were recorded in the permanent dentition while data on confounding variables were collected through questionnaires. Caries increment and progression were the outcome variables. A total of 408 children participated in the baseline examination, 208 finished the study. After adjusting for confounders, the prevented fraction (PF) on occlusal surfaces for advanced caries in the experimental group was 76.1% (mean difference- −0.35, 95% CI: −0.67–0.04), while the PF for progression from incipient to advanced caries was 100%(mean difference- −0.30, 95% CI: −0.52–0.09). The mean number of smooth surfaces that progressed from incipient to advanced caries in the comparison group was more than twice that of the experimental group, the mean difference was −0.25 (95% CI: −0.46–−0.03) with a PF of 61%. The intervention was only effective in preventing advanced caries on occlusal surfaces and in halting the progression of caries on occlusal and smooth surfaces but not on approximal caries.

## 1. Introduction

Dental caries still constitutes a public health challenge with more than half a billion of the global child population estimated to have untreated caries in their deciduous dentition [1]. Data in the Australian context indicate that while there was a steady decline in the caries burden in the deciduous dentition of children between the mid-1970s and 1990s, a gradual rise has been reported after the mid-1990s [2]. Further, the oral disease burden among Indigenous children, especially those living in remote regions, is exceptionally high [3].

The Australian National Child Oral Health survey 2012–2014 found that the number of untreated, permanent, decayed teeth in Indigenous children aged 6–14 years were on average 0.7, nearly three times greater than in non-Indigenous children; among the Indigenous children, those living in remote/very remote regions had four times greater number of untreated decayed teeth than those from major cities [3]. More Indigenous children (31%) reported less favorable visiting patterns when compared to other Australian children (20%) [4]. This difference can be attributed partly to the lower access to care among remote Indigenous communities, where the dentist to population ratio is 23:100,000 people compared to 72:100,000 in major cities [4]. Particularly in the remote Northern Peninsula Area (NPA) of Far North Queensland (FNQ) region, we found that the children aged 4–17 years had on average 3.64 surfaces with tooth decay in the deciduous and permanent dentition [5]. There has been a gradual increase in caries burden in this population, particularly after the water fluoridation was withdrawn in 2011 [6,7].

Active interventions such as professionally applied fluorides and fissures sealants could help alleviate the increasing caries burden in this population that do not have water fluoridation, nor access to regular dental services. Fluoride varnishes (FV) reduce the caries experience in children’s permanent and deciduous dentitions by 43% and 37%, respectively [8]. The addition of an antimicrobial, such as povidone–iodine (PI), was recently found to enhance the caries prevention efficacy of FV [9]. Additionally, a Cochrane review reported that fissure sealants (FS) resulted in caries reductions of 11–51% on permanent occlusal surfaces in children and adolescents two years after application [10]. Therefore, an intervention strategy, comprising of FS to prevent caries on the occlusal surfaces [11], as well as PI and FV applied once annually for two consecutive years, was undertaken [12]. Although the Queensland Health Department and other evidence-based literature recommend two applications of FV per year [13,14], we wished to investigate the efficacy of a single annual application, provided by clinicians on a fly-in/fly-out basis, as the costs of two visits in a single year from a dental team would be prohibitive.

To the authors’ knowledge, there are no studies that have compared the effectiveness of such interventions on specific tooth surfaces. This study aimed to evaluate the effect of two annual interventions of FS, PI, and FV in preventing caries on occlusal, approximal, and smooth tooth surfaces of children in a remote Indigenous community.

## 2. Material and Methods

The Human Research Ethics Committees (HREC) of Griffith University (DOH/05/15/HREC) and Far North Queensland (HREC/15QCH/39-970) approved this study. A site-specific agreement was established with the Torres and Cape Hospital and Health Service, and formal permissions were obtained from the Department of Education and Training of the Queensland Government for access to children on school premises. This intervention study is registered with the Australian New Zealand Clinical Trials Registry, ACTRN12615000693527, and the protocol has been published [12].

### 2.1. Participants

This non-randomized clinical trial was conducted in the NPA of FNQ, Australia. All children (approximately 600) attending the three schools in the NPA were invited to participate. As all the children were eligible, a probability sampling technique could not be adopted. Two informed consent processes were involved: consent was sought from parents/guardians for an interview-based questionnaire and epidemiological examination of the head, neck, and oral cavity; and a separate consent for restorative treatment of any existing dental disease, followed by the preventive intervention. Most children, (*n* = 408), received consent for the epidemiological examination, and these constituted the total study sample. Consent for the treatment of existing disease and the preventive intervention could not be obtained for approximately half of the study sample, and these constituted a comparison group. Details on the study location and methodology have been previously published [12]. Baseline examinations, dental treatment, and the preventive intervention were provided in October 2015 followed by re-examination and re-application of the intervention in October 2016 and 2017.

### 2.2. Data Collection

Children in both the intervention and comparison groups were interviewed at baseline to record age, sex, sugar consumption (soft drinks, fruit juices, sweets and lollies, syrups/jams/spreads, adding sugar to beverages) on a typical day with a dichotomous response (yes/no), as well as toothbrushing (‘twice or more a day’ and ‘once or less a day’). Stimulated saliva was collected over 5 min by chewing on paraffin wax to assess flow rate in mL/5 min, pH, and total buffering capacity which were used as continuous measures in the statistical analysis (17). Chairside kits were used to assess levels of *mutans streptococci* (MS) and *Lactobacilli* (LB). Children with salivary MS and LB levels of ≥ 10^5^ colony forming units (CFU)/mL saliva were considered ‘high risk’, while those with < 10^5^ CFU/mL saliva were classified as ‘low risk’ as recommended by the manufacturer [5,12].

Dental disease was scored using the International Caries Detection and Assessment System (ICDAS) [15] on all three occasions, by four calibrated examiners who were experienced dentists/oral health therapists. All examiners completed the online training module for ICDAS and underwent calibration exercises against the chief investigator at baseline which involved examining ten children and discussing the discrepancies. To assess the agreement between examiners, 5% of children examined by one examiner were re-examined by another. Any discrepancies in the assessment were discussed to prevent future disagreements. ICDAS criteria involve a two-digit coding system for each tooth surface. The first records presence and type of any restoration present, the second records the caries status. For the latter, a score of ‘0’ represents a sound surface; codes 1 and 2 indicate a visual change in enamel without cavitation; and codes 3–6 record enamel breakdown without or with dentine involvement. ICDAS has been found to demonstrate high reproducibility and accuracy for caries evaluation on coronal surfaces [15]. In the present study, scores 1 and 2 were considered as incipient, and 3–6 as advanced caries, thus aligning with the World Health Organization’s caries criteria [16]. Only the permanent dentition is considered in the present analyses as most children had mixed dentitions with many deciduous teeth at the stage of exfoliation.

The clinical assessment involved visual–tactile examination conducted in schools on portable dental chairs under strong artificial illumination using disposable dental mirrors and blunt probes. No radiographs were taken. Although the examiners were not informed of the case/comparison status of the children, the presence of fissure sealants as part of the intervention made masking impractical.

### 2.3. Interventions

During the first visit, consented children in need in the experimental group were rendered dentally fit by a team of trained oral health professionals in the local hospital’s dental clinic or a mobile dental van. All then had fluoride-releasing FS (Conseal F: SDI Limited, Bayswater, Victoria, Australia) placed in sound pits and fissures on occlusal surfaces of erupted premolars and molars. Manufacturer’s instructions were followed, involving cleaning with prophylaxis paste, isolation of the teeth, etching of the surfaces, then application of FS using manufacturer-provided syringes and light-curing. For applying PI 10%, pre-packed swab sticks were used (PDI^®^ PVP Iodine swab sticks: Professional Disposables International Inc., Orangeburg, NY, USA) to cover all tooth surfaces. After washing and drying, a pre-packed single dose of 0.4 mL (0.4 g of 22,600 parts per million fluoride) sodium fluoride varnish (Duraphat^®^: Colgate-Palmolive Pty Ltd, Sydney, NSW, Australia) was applied to all tooth surfaces using the brush provided by the manufacturer.

Delivery of the preventive intervention to each child took approximately 10 min. A similar protocol was adopted at the follow-ups, where any surfaces with lost fissure sealants were re-sealed. The intervention group children also received active treatment when required. Conversely, children in the comparison group did not receive any intervention other than the usual care, which is mostly emergency care. All children received a fluoridated toothpaste and soft-bristled toothbrush at each appointment. No other incentives were offered.

### 2.4. Statistical Methods

SPSS (IBM Corp, Armonk, NY, USA) was used for statistical analyses. Caries increment in the permanent dentition after two years, defined as the total number of surfaces with new caries on occlusal, approximal and smooth surfaces, and caries progression, were the outcomes of interest. Caries progression was assessed by enumerating the number of surfaces with initial caries (ICDAS scores 1 and 2) at baseline that had progressed to advanced caries (ICDAS scores 3–6) at two-year follow-up. Caries increment was quantified separately for incipient (surfaces that were caries-free at baseline but presenting ICDAS scores 1 and 2 at two-years follow-up) and advanced caries (surfaces that were caries-free at baseline but presenting with ICDAS scores 3–6 at two-years follow-up).

A Fisher exact test and unpaired *t*-tests were used to compare the categorical and continuous baseline characteristics respectively between the experimental and comparison groups. Caries incidence was not considered an appropriate outcome as most children had one or more surfaces with caries. An unpaired *t*-test was used to assess the differences in mean surfaces with new caries or progression between groups.

To assess the efficacy of the intervention on caries increment and progression, general linear regression models were used. Estimated marginal means from the models provided mean values and 95% confidence intervals (CI) for caries increment and progression in the experimental and comparison groups after adjusting for confounding variables of age, sex, salivary factors, sugar consumption, and tooth brushing. Mean difference with 95% CI between the experimental and comparison groups and caries Prevented Fraction (PF) were also estimated to demonstrate intervention efficacy. PF was calculated as the difference in mean caries increment/progression between groups expressed as a percentage of mean increment/progression in the comparison group.

Data are only available upon request due to ethical considerations regarding the Indigenous communities in the study. Access to raw data may be requested from the corresponding author n.johnson@griffith.edu.au or from the Griffith University Human Research Ethics Committee (Ref: DOH/05/15/HREC) at research-ethics@griffith.edu.au.

## 3. Results

A total of 408 children participated in the baseline examination: 196 received the intervention and the remaining 212 constituted the comparison group. At the end of the two years, 117 and 91 children remained in experimental and comparison groups respectively. The mean age of the children at baseline was 8.66 years (range: 4–17). There were more females in both experimental (55.2%) and comparison (53.3%) groups. There were no significant differences between the groups for most demographic, behavioral, and salivary characteristics at baseline (Table 1). However, more children in the comparison group (78.9%) reported adding sugar to their cereals/tea/coffee than the experimental group (67.3%). Also, significantly more children in the comparison group (79.2%) had higher salivary loads (≥ 10^5^ CFU/mL) of MS than the experimental group (67.6%). The overall Kappa statistic for inter-examiner agreement for caries assessment was 0.84.

Table 2 shows that the PF of incipient and advanced caries on occlusal surfaces in the experimental group was 31.6% and 55% respectively. The mean difference in caries increment between the experimental and comparison groups for incipient and advanced caries on the occlusal surfaces was −0.56 (95% CI: −0.03–−1.08) and −0.22 (95% CI: −0.03–−0.41) respectively. There were similar findings on smooth surfaces, with advanced caries increment in the comparison group being approximately twice that of the experimental group (mean difference- −0.20, 95% CI: −0.01–−0.40 and PF- 52.6%). The intervention was effective in halting the caries progression on smooth surfaces, mean difference was −0.17 (95% CI: −0.01–−0.34) and the PF was 53.1. Caries increment on the approximal surfaces was not significantly different between the groups.

Table 3 demonstrates that the adjusted increment of advanced caries on the occlusal surfaces of the comparison group was approximately four times of that observed in the experimental group (mean difference- −0.35, 95% CI: −0.67–−0.04) with a PF of 76.1%. Further, there were some reversals in the experimental group in terms of caries progression on the occlusal surfaces, while the mean occlusal surfaces that showed caries progression in the comparison group was 0.29 (95% CI: 0.02–0.55) resulting in a mean difference of −0.30 (95% CI: −0.52–−0.09). Mean caries progression on smooth surfaces in the experimental group was 0.16 (95% CI: −0.07–0.40) and 0.41 (95% CI: 0.14–0.67) in the comparison group, resulting in a mean difference of −0.25 (95% CI: −0.46–−0.03) and a PF of 61%.

## 4. Discussion

This annual intervention provided over two consecutive years comprising of the application of FS, PI, and FV was effective in preventing advanced caries lesions on occlusal surfaces only. It was also effective in halting the progression of caries from incipient to advanced in both occlusal and smooth surfaces of the permanent dentition at the end of two years.

Other studies from Australia with Indigenous children have found FV to be effective on the deciduous dentition [17], but the effect on the permanent dentition is not widely reported from Australia, although several studies conducted elsewhere have shown benefits to the permanent dentition [18]. Though more frequent application of FV is recommended (usually biannual) [19], one annual application was found to have a modest caries protective effect [20]. With a five-year retention rate between 65% and 84% for different fissure sealant materials, these clearly play a significant role in preventing caries on occlusal surfaces [21]. Recently, FS has also been recommended in controlling initial tooth decay not only on occlusal but also approximal surfaces [10,22]. As PI could limit the binding potential of MS to tooth surfaces by inhibiting the enzyme required to produce extracellular polysaccharides [9], it has been gaining importance recently in caries prevention. Results from an experimental study demonstrated that the usage of PI along with FV was beneficial over the use of FV alone [9].

To the authors’ knowledge, this is the first study that has evaluated the combined effect of FS, PI, and FV. We could find only one study that used a combination of FS and FV, which concluded that the combination was more effective than FV alone [23]. A Federal Government funded project “Futures in the Northern Territory Health Implementation Plan Oral Health Program” administered an annual application of FV and FS, and also treatment services, to Indigenous children. Although there was no comparison group, as this was delivered to all children, and no attempt was made to specifically evaluate the effects, the data demonstrate subsequent reductions in the proportion of children with dental decay [24].

In the present study, we observed that the combination of FS, PI, and FV was not effective in preventing dental caries or halting caries progression on approximal surfaces of the permanent dentition. As no attempt was made to seal approximal surfaces, any caries preventive effect on approximal surfaces could be attributed to PI and FV alone. While there is no literature on the effect of PI specifically on approximal caries, there is limited but conflicting evidence on the effectiveness of FV on approximal caries. Swedish studies have found a biannual school-based fluoride varnish program to be effective in preventing and inhibiting the progression of approximal caries after three years in children with high caries levels [25,26], but not effective in a 3.5 year study for children with low levels of disease [27]. In contrast, another study from Sweden, conducted more than three decades ago, found FV to be effective in preventing approximal caries in children with low or moderate caries activity, but not in those with high activity [28]. Direct comparison with the results of this study is limited by slight differences in protocols. The Swedish studies flossed interdental areas before applying varnish (which we did not), and sometimes used a syringe to inject varnish between teeth (whereas we used brushes). The brush requires frequent reloading of the FV, which we found led to limited cooperation from the children; syringes may be preferable [29]. Some clinicians also recommend flossing with FV to enable the varnish to reach the approximal surfaces [30,31]. Research related to direct comparisons between modes of application (brush or syringe or cotton pellets or floss) of FV was not found, but there are studies that have evaluated the influence of oral prophylaxis on the effectiveness of FV, and these have concluded that prophylaxis does not alter the caries preventive potential of FV [8]. Further studies of method of application of FV are warranted.

Although some believe that fluorides (including varnishes) are more effective in preventing caries on smooth surfaces than the occlusal surfaces [32,33], a report by McDonald and Sheiham that combined the data from 18 epidemiological studies contradicts this perspective [34]. In this study, we found that the intervention was ineffective in preventing caries on the smooth surfaces. Nevertheless, it was effective in halting the progression of smooth surface caries. From the literature published decades ago, there is conflicting evidence on the efficacy of fluoride varnish on smooth surfaces of the permanent dentition, with one study finding no effect on buccolingual surfaces [35] yet another finding the effect on smooth surfaces decreasing when adjusted for confounding variables [36]. More laboratory and experimental studies are required to ascertain the surface-specific effect of fluoride varnish.

Limitations of the present study include small sample size, high attrition, and lack of a true control group; a randomized control design was not possible for ethical reasons. Moreover, it was not possible to evaluate the efficacy of each component separately due to the use of a single intervention that combined three proven caries prevention agents.

## 5. Conclusions

In conclusion, this intervention protocol was effective in preventing new incipient and advanced caries on occlusal surfaces, but not on approximal or smooth surface caries. Fewer occlusal and smooth surfaces with incipient caries progressed to advanced caries in the experimental than the comparison group. However, the differences for caries increment or progression between the experimental and comparison groups were not significant for approximal surfaces.

## Figures and Tables

**Table 1:** Demographic, behavioral, and salivary characteristics in experimental and comparisons groups at baseline (*N* = 408).

Background Characteristics		Experimental	Comparison Group	Significance
		*n* (%)	*n* (%)	
Sex *	*Males*	87 (44.8)	98 (46.7)	0.764
	*Females*	107 (55.2)	112 (53.3)	
On a typical day, do you drink soft drinks *	*Yes*	125 (72.3)	164 (80.8)	0.065
	*No*	48 (27.7)	39 (19.2)	
On a typical day, do you consume fruit juice *	*Yes*	162 (92.6)	193 (94.6)	0.527
	*No*	13 (7.4)	11 (5.4)	
On a typical day, do you consume sweets and lollies *	*Yes*	146 (84.4)	180 (88.7)	0.228
	*No*	27 (15.6)	23 (11.3)	
On a typical day, do you consume syrups, jams, and sweet spread *	*Yes*	150 (87.7)	189 (93.1)	0.108
	*No*	21 (12.3)	14 (6.9)	
Do you add sugar to your cereal, tea, coffee, or milo *	*Yes*	115 (67.3)	161 (78.9)	**0.013**
	*No*	56 (32.7)	43 (21.1)	
How often do you brush your teeth *	*Once or less than once a day*	38 (22.9)	55 (27.2)	0.399
	*Twice or more a day*	128 (77.1)	147 (72.8)	
**Salivary pH** Mean (SD) §		7.07 (0.44)	7.07 (0.52)	0.672
**Stimulated salivary flow/5 min** Mean (SD) §		6.15 (3.20)	5.82 (3.08)	0.533
**Total buffering capacity** Mean (SD) §		9.46 (2.02)	9.1 (2.1)	0.161
Mutans Streptococci levels *	*Low risk*	56 (32.4)	22 (20.8)	**0.040**
	*High risk*	117 (67.6)	84 (79.2)	
**Lactobacilli** levels *	*Low risk*	112 (64.7)	56 (52.8)	0.059
	*High risk*	61 (35.3)	50 (47.2)	
**Age** Mean (SD) §		8.73 (3.23)	8.59 (3.23)	0.661

Total number of subjects for most variables does not equal to 408 due to missing values; * Fisher exact test; § Unpaired *t*-test; *p*-values < 0.05 are in bold.

**Table 2:** Mean caries increment on different surfaces of the permanent dentition in the experimental and comparison groups after two years.

Caries outcome	Experimental (95% CI)	Comparison (95% CI)	Mean Difference (95% CI)	Prevented Fraction (%)	*p*-Value
**Caries increment on occlusal surfaces**					
Incipient caries	1.21 (0.94–1.49)	1.77 (1.32–2.22)	−0.56 (−0.03–1.08)	31.6	**0.029**
Advanced caries	0.18 (0.08–0.28)	0.40 (0.23–0.56)	−0.22 (−0.03–0.41)	55.0	**0.020**
Progression from incipient to advanced	0.08 (0.02–0.14)	0.20 (0.08–0.32)	−0.12 (−0.26–0.01)	60.0	0.059
**Caries increment on approximal surfaces**					
Incipient caries	1.50 (1.0–1.94)	1.59 (1.14–2.04)	−0.10 (−0.74–0.54)	5.6	0.764
Advanced caries	0.19 (0.08–0.30)	0.18 (0.06–0.29)	0.01 (−0.15–0.17)	–5.6	0.882
Progression from incipient to advanced	0.06 (0.004–0.12)	0.10 (0.02–0.18)	−0.04 (−0.13–0.06)	40	0.420
**Caries increment on smooth surfaces**					
Incipient caries	1.65 (1.24–2.06)	1.99 (1.61–2.36)	−0.34 (−0.90–0.23)	17.1	0.237
Advanced caries	0.18 (0.08–0.28)	0.38 (0.22–0.55)	−0.20 (−0.01–0.40)	52.6	**0.028**
Progression from incipient to advanced	0.15 (0.06–0.23)	0.32 (0.16–0.47)	−0.17 (−0.01–0.34)	53.1	**0.037**

Unpaired *t*-test, *p*-values < 0.05 are in bold.

**Table 3:** Adjusted caries increment on different surfaces of the permanent dentition in the experimental and comparison group at two years follow-up.

Caries Outcome	Experimental (95% CI)	Comparison (95% CI)	Mean Difference (95% CI)	Prevented Fraction (%)	*p*-Value
**Caries increment on occlusal surfaces**					
Incipient caries	1.71 (0.86–2.56)	2.20 (1.24–3.16)	−0.49 (−1.26–0.28)	22.3	0.212
Advanced caries	0.11 (−0.24–0.46)	0.46 (0.07–0.85)	−0.35 (−0.67–0.04)	76.1	**0.030**
Progression from incipient to advanced	−0.02 (−0.26–0.22)	0.29 (0.02–0.55)	−0.30 (−0.52–0.09)	100	**0.006**
**Caries increment on approximal surfaces**					
Incipient caries	2.31 (1.23–3.39)	2.55 (1.34–3.76)	−0.24 (−1.22–0.74)	9.4	0.626
Advanced caries	0.17 (−0.07–0.41)	0.05 (−0.22–0.32)	0.12 (−0.01–0.34)	−70.6	0.277
Progression from incipient to advanced	0.07 (−0.04–0.17)	0.02 (−0.01–0.13)	0.05 (−0.04–0.14)	−71.4	0.302
**Caries increment on smooth surfaces**					
Incipient caries	1.77 (0.79–2.75)	2.46 (1.36–3.56)	−0.69 (−1.58–0.20)	28	0.129
Advanced caries	0.25 (−0.07–0.56)	0.44 (0.08–0.80)	−0.19 (−0.48–0.10)	43.2	0.194
Progression from incipient to advanced	0.16 (−0.07–0.40)	0.41 (0.14–0.67)	−0.25 (−0.46–0.03)	61	**0.025**

General linear regression model, *p*-values < 0.05 are in bold. Adjusted for baseline sex, age, daily consumption of soft drinks, fruit juices, sweets and lollies, syrups/jams/spreads, sugar with milk, twice or more tooth brushing, salivary pH, stimulated salivary flow rate, total buffering capacity, salivary MS, and LB levels.
